# Acceptability of artificial intelligence in inclusive education: a TAM2-based study among preservice teachers

**DOI:** 10.3389/frai.2025.1616327

**Published:** 2025-08-22

**Authors:** Houda Amouri, Samia Haroud, Lynda Ouchaouka, Nadia Saqri

**Affiliations:** ^1^Multidisciplinary Laboratory in Education Sciences and Training Engineering (LMSEIF), Hassan II University of Casablanca, Casablanca, Morocco; ^2^Mathematics, Artificial Intelligence, and Digital Learning Laboratory (Mind-Lab), Hassan II University of Casablanca, Casablanca, Morocco

**Keywords:** artificial intelligence, technology acceptance model, ADHD, inclusive education, pre-service teachers

## Abstract

**Introduction:**

The integration of artificial intelligence (AI) into education is generating growing interest, particularly due to its potential to support inclusive pedagogical practices. This is especially relevant for addressing the needs of students with attention deficit hyperactivity disorder (ADHD). The success of such integration largely depends on the acceptability of AI tools by educators, especially those still in initial training. This study aims to identify the factors influencing the acceptability of AI among pre-service teachers in the specific context of teaching students with ADHD.

**Methods:**

Grounded in the Technology Acceptance Model 2 (TAM 2), the study adopts a mixed-methods approach. Quantitative data were collected via structured questionnaires, and qualitative insights were obtained through semi-structured interviews with pre-service teachers in Morocco.

**Results:**

Findings reveal that perceived usefulness is the most influential predictor of AI acceptability, followed by perceived ease of use, voluntariness, and subjective norms. Participants emphasized the potential of AI to enhance pedagogical efficiency and support differentiated instruction. Institutional support and interface simplicity also emerged as key enablers.

**Discussion/Conclusion:**

These results highlight the need to incorporate digital literacy into teacher training programs and to develop AI tools specifically adapted to students with special educational needs. They also call for the establishment of a robust ethical and regulatory framework to ensure the responsible, equitable, and secure use of AI in education.

## 1 Introduction

The integration of digital technologies in education has deeply changed teaching practices in recent decades. Among these innovations, artificial intelligence (AI) is playing an increasingly central role. It offers tools that can personalize learning, automate administrative tasks, and support teachers. This is especially helpful for assisting students with special educational needs (Zawacki-Richter et al., 2019; Fadel et al., 2019).

Among these students, those with attention deficit hyperactivity disorder (ADHD) are particularly concerned. This disorder is recognized by the American Psychiatric Association (APA) as one of the most commonly diagnosed neurodevelopmental disorders. It affects about 8.4% of children and 2.5% of adults worldwide. These numbers show the scale of the issue and highlight the urgent need to create appropriate educational resources. This is especially important in contexts like Morocco, where support systems for students with special needs are still developing.

Several recent studies have shown the potential of AI to meet the specific needs of these students. This includes adapting content, teaching pace, and learning methods (Barua et al., 2022; Moleka, 2023). These adaptations are made possible through innovative technologies (Richter et al., 2023) or targeted mobile applications (Kyriakaki and Driga, 2023).

In this context, AI appears to be a powerful tool for promoting school inclusion. It can also improve the effectiveness of differentiated teaching practices. However, its actual use by teachers depends on several factors. Beyond simple access to technology, its acceptability relies on individual variables. These include experience with digital tools, trust in their use, and willingness to try new approaches (Cukurova et al., 2023). Contextual factors also play a role, such as training received and institutional support (Jabraoui and Vandapuye, 2024; Holstein et al., 2019).

In this perspective, theoretical models of technology acceptance, especially the Technology Acceptance Model (TAM) (Venkatesh and Davis, 2000) and the UTAUT model (Venkatesh et al., 2003)—offer useful frameworks to analyze AI adoption. These models highlight key variables such as perceived usefulness, ease of use, social influence, and institutional norms.

This study follows that framework. Its main goal is to examine the factors that influence the acceptability of AI tools in teaching students with ADHD. It focuses on how future teachers perceive these technologies. It also explores how different factors—such as perceived usefulness, ease of use, personal experience, willingness, and social and institutional norms—affect their intention to use Ai in their teaching practices. In this study, acceptability is defined as the intention to adopt AI tools in future teaching practices, rather than as a general attitude. This definition is consistent with the Technology Acceptance Model 2 (TAM2), where behavioral intention serves as the main predictor of actual technology use (Venkatesh and Davis, 2000).

This research is guided by three main questions:

1. What are future teachers’ general perceptions of the different factors influencing the acceptability of AI for teaching students with ADHD?
2. Which factors have the strongest influence on this acceptability?
3. Which item is the most decisive in the intention to adopt AI in this specific context?

## 2 Theoretical framework

To analyze the factors that influence the acceptability of artificial intelligence in education, this study is based on the Technology Acceptance Model (TAM), as extended by Venkatesh and Davis (2000) through TAM 2. This model, widely used in education and technology innovation research, states that the intention to use technology is mainly influenced by two core beliefs: perceived usefulness (the expected benefits of the technology) and perceived ease of use (how simple the tool is to use). TAM 2 expands the original model by adding social and cognitive factors that may influence technology acceptance. In our study, five dimensions were selected. These were chosen for their theoretical relevance and their suitability to the profile of the respondents, who are future teachers in initial training: perceived usefulness, which reflects how effective AI is seen in improving teaching for students with ADHD; perceived ease of use, which measures how accessible and user-friendly AI tools are perceived to be; and subjective norm, referring to the perceived influence of the professional environment—such as trainers, peers, and institutions—on the use of AI. In addition, social influence captures how AI adoption is viewed in terms of professional value and recognition, while experience and willingness encompass both prior experience with digital tools and the intrinsic motivation to adopt innovative technologies. These constructs are drawn from the Technology Acceptance Model and its extensions (Davis, 1989; Venkatesh and Davis, 2000).

Some specific dimensions from TAM 2—such as job relevance, output quality, and result demonstrability—were intentionally excluded. These variables require concrete and extended experience with the technology, which most participants in this study, still in initial training, do nothave. Thus, the theoretical framework was adapted to fit the study context and the participants’ level of exposure to artificial intelligence.

## 3 Materials and methods

This study uses a mixed-methods approach, combining both quantitative and qualitative methods. The goal is to identify the factors that influence future teachers’ acceptance of integrating artificial intelligence (AI) tools into their teaching for students with attention deficit hyperactivity disorder (ADHD).

### 3.1 Quantitative analysis

**Figure 1:**
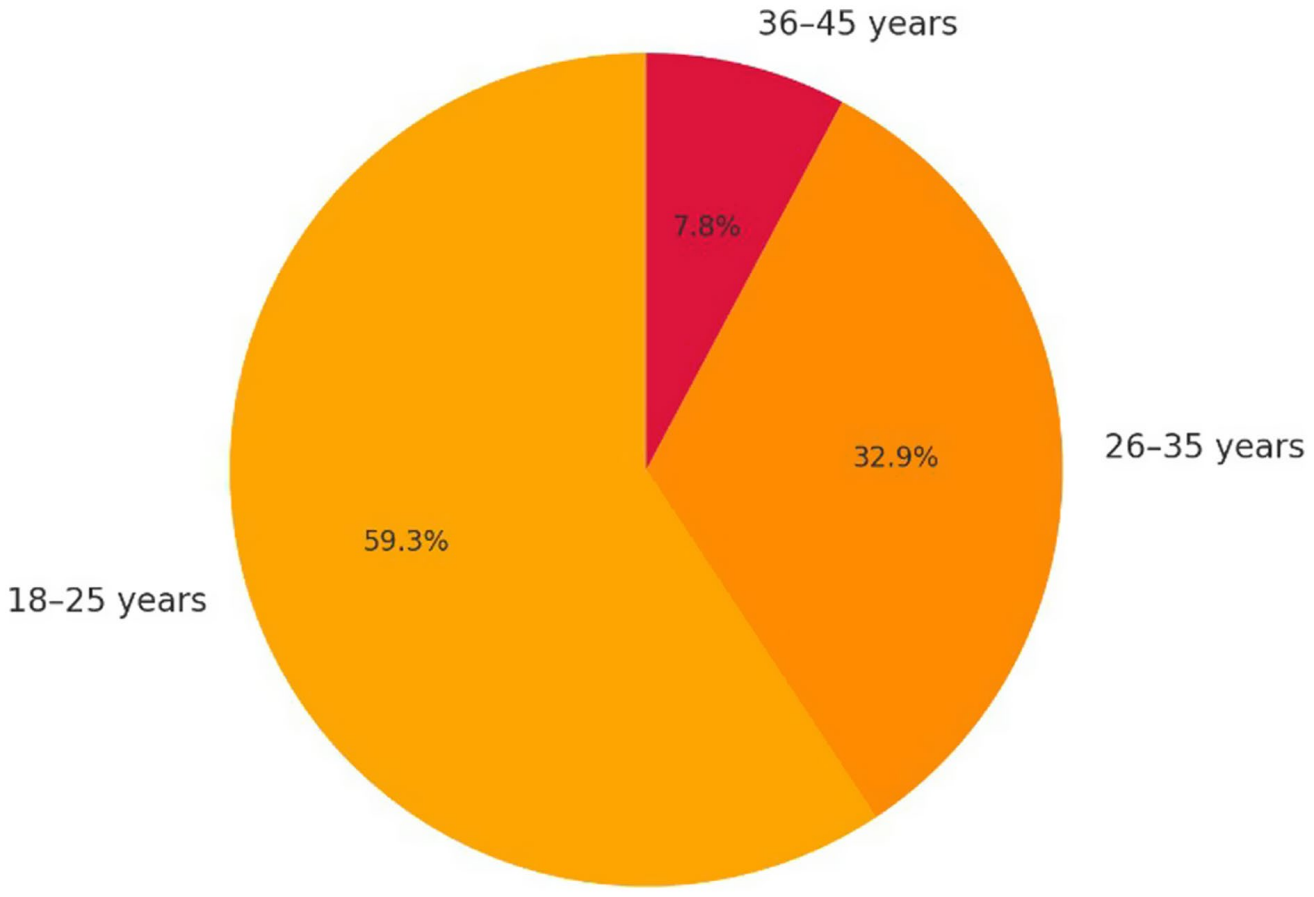
Age distribution of participants.

Data were collected using a structured questionnaire grounded in established theoretical models of technology acceptance, particularly the Technology Acceptance Model 2 (TAM2) developed by Venkatesh and Davis (2000). The questionnaire comprised multiple sections, some focusing on participants’ sociodemographic characteristics and others targeting key factors influencing technology acceptance. The assessed dimensions included perceived usefulness, perceived ease of use, subjective norms, social influence, experience, willingness, and intention to use. Each construct was measured using two to three items, selected based on previous TAM-related studies to ensure content validity. Participants responded on a 4-point Likert scale ranging from 1 (“strongly disagree”) to 4 (“strongly agree”). The original version of the questionnaire was developed in English and then translated into French to enhance comprehension while preserving the meaning and integrity of the items. Internal consistency was evaluated using Cronbach’s alpha, with all constructs demonstrating acceptable to good reliability (*α* = 0.74–0.83), indicating that the items within each dimension were sufficiently correlated to be considered reliable measures of the corresponding latent variables. TAM2 has been widely used and validated in various domains, including education, to assess technology adoption (Venkatesh et al., 2003; Venkatesh and Bala, 2008) (Figure 1).

### 3.2 Qualitative analysis

To enrich the quantitative data, an open-ended section was added after each part of the questionnaire. Each section was linked to a specific factor of acceptability. These open sections allowed respondents to share additional observations or mention other influencing factors. This approach aimed to better understanding elements that may affect the acceptance of AI in teaching students with ADHD.

The qualitative responses were analyzed using Bardin’s (2001) content analysis method. This includes three main steps: pre-analysis, data coding, and information processing. This method made it possible to identify recurring themes and patterns in participants’ comments. It provided an interpretive complement to the quantitative results and offered deeper insight into future teachers’ perceptions of AI.

### 3.3 Sample

The sample consists of 164 preservice teachers enrolled in initial teacher training at the Higher Normal School of Casablanca. Among the participants, 87.2% (*n =* 143) are women and 12.8% (*n =* 21) are men. Participants’ ages vary: 58.7% are between 18 and 25 years old, 32.6% between 26 and 35, and 7.7% between 36 and 45.

**Figure 2:**
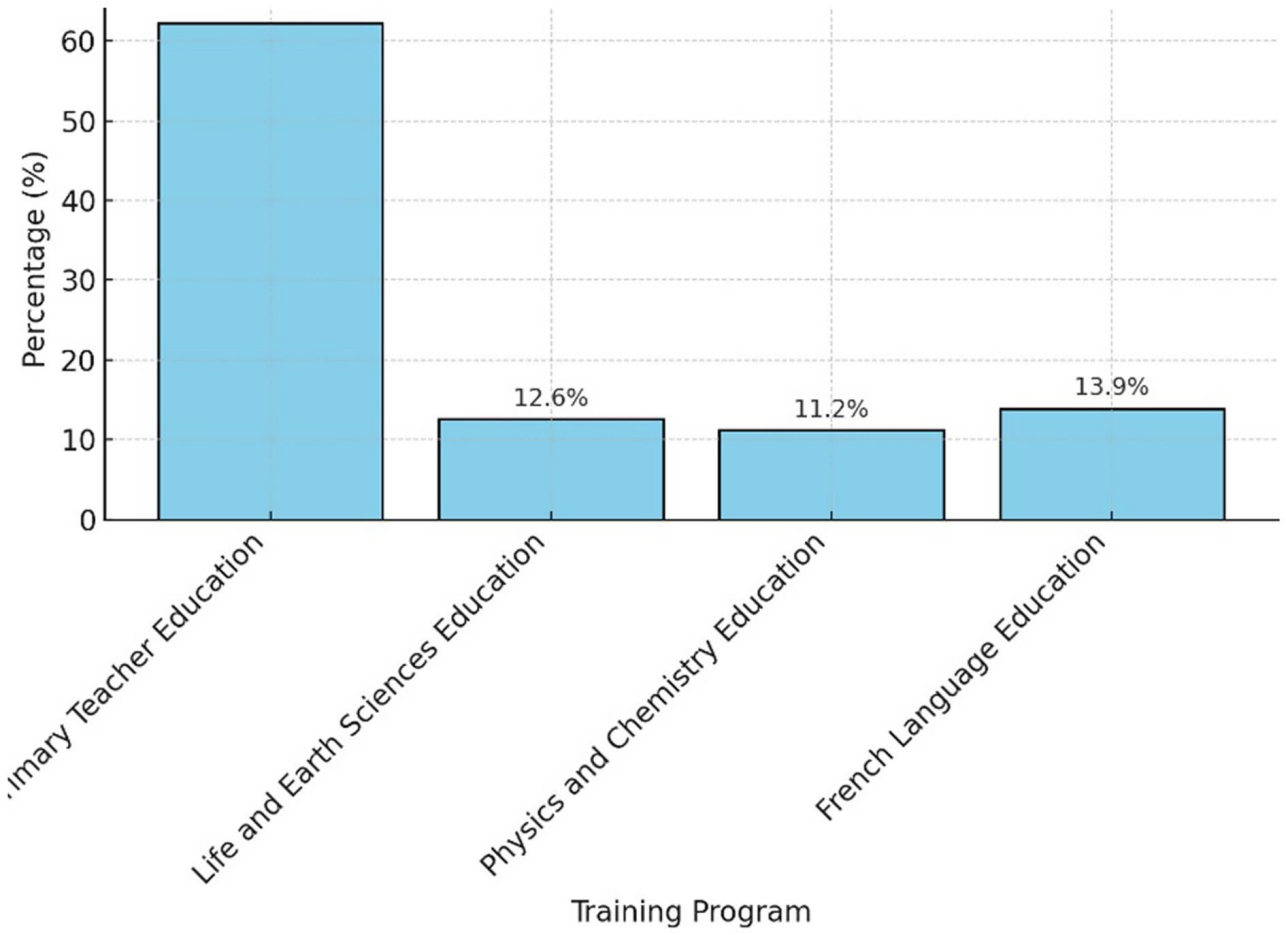
Distribution of participants by training program.

Participants come from different training programs (Figure 2). About 62.3% are in primary education, 12.6% in life and earth sciences, 11.2% in physics and chemistry, and 13.9% in French language teaching.

Out of the 248 students contacted, 164 completed the questionnaire, resulting in a response rate of 66.1%. Prior to participation, all respondents received an informed consent form along with a detailed explanation of the study’s objectives and procedures.

Regarding data quality, incomplete responses (*n =* 35) were excluded from the final dataset using case-wise deletion (also known as listwise exclusion). Only fully completed questionnaires were retained for analysis. To address potential sample selection bias, available sociodemographic characteristics—specifically age and gender of respondents were compared to those of the broader cohort of preservice teachers at the institution. No significant deviations were observed, suggesting acceptable representativeness with respect to these variables.

## 4 Results

### 4.1 Descriptive analysis of future teachers’ perceptions of AI

The analysis is based on descriptive statistics for the different measured dimensions, including the mean score and standard deviation (SD). These statistics provide an initial overview of general trends and the variability in participants’ responses (*N =* 164).

The analysis of descriptive statistics reveals contrasting trends in the acceptability of AI (Table 1). Among the studied factors, social influence shows the highest mean score (M = 2.87, SD = 0.958), indicating that teachers are strongly influenced by the opinions of colleagues and experts. In contrast, perceived ease of use has the lowest mean score (M = 2.56, SD = 1.000), suggesting that perceived complexity may hinder adoption. Subjective norms (M = 2.59, SD = 1.022) reflect a lack of clear institutional support, while perceived usefulness (M = 2.74, SD = 0.999) and prior experience with similar tools (M = 2.70, SD = 1.056) are evaluated as moderate influences.

**Table 1:** Descriptive statistics of the analyzed dimensions (*N =* 164).

Section	*N*	Mean score	Standard deviation (SD)
Perceived usefulness	164	2.74	0.999
Experience and Willingness	164	2.70	1.056
Perceived Ease of Use	164	2.56	1.000
Social influence	164	2.87	0.958
Subjective norm	164	2.59	1.0225

The descriptive analysis helped identify general trends in teachers’ perceptions of artificial intelligence (AI). However, these initial observations do not establish which variables actually influence the intention to adopt AI, which is the dependent variable of this study. To explore this further, a multiple linear regression was conducted to determine the impact of the various factors on this intention.

### 4.2 Factors of artificial intelligence (AI) acceptability among future teachers for ADHD education

The results presented in Table 2 highlight the relative weight of each factor in predicting future teachers’ intention to adopt AI for teaching students with ADHD.

**Table 2:** Analysis of factors influencing AI acceptance—regression coefficients and statistical significance (*p*-value).

Factor	Coefficient (*β*)	SE	*P*-value
Social influence	0.2256	0.064	< 0.001
Perceived ease of use	0.2543	0.063	< 0.001
Subjective norm	0.1888	0.061	< 0.001
Perceived usefulness	0.6139	0.045	< 0.001
Experience and willingness	0.2572	0.062	< 0.001

The results (Table 2) indicate that the model is statistically significant (*p <* 0.001) and explains 29.8% of the variance in the intention to adopt AI (*R^2^* = 0.298). This level of explained variance suggests that, although the variables included in the model make a meaningful contribution, other factors not covered in this study may also play a key role in AI adoption.

An examination of the regression coefficients (*β*) shows that perceived usefulness (*β* = 0.6139, SE = 0.063, *p <* 0.001) has the strongest impact on the intention to adopt AI. This finding highlights the importance of teachers’ perceptions of the benefits of AI for their teaching. The more teachers perceive AI as useful, the more likely they are to intend to adopt it.

Other variables also show a significant effect, though to a lesser extent. Experience and willingness (*β* = 0.2572, SE = 0.062, *p <* 0.001) and perceived ease of use (*β* = 0.2543, SE = 0.063, *p <* 0.001) have comparable impacts. This suggests that teachers who are more familiar with new technologies, or who perceive AI as intuitive and accessible, are more likely to adopt it.

Social influence (*β* = 0.2256, SE = 0.064, *p <* 0.001) and subjective norm (*β* = 0.1888, SE = 0.061, *p <* 0.001) also contribute, albeit less prominently. These results indicate that encouragement from the professional environment, as well as perceived social expectations, influence the intention to adopt AI though to a lesser degree than practical or perceived benefits.

In conclusion, this analysis shows that perceived usefulness is the primary driver of AI acceptability among future teachers, followed by practical factors such as experience and ease of use.

To better understand how these factors operate, an item-level analysis was conducted. However, although the overall effects of perceived usefulness and experience/willingness were significant, their individual items did not show significant effects and were thus excluded from the item-level breakdown. The following sections present detailed analyses of the key items within the other influential factors.

### 4.3 Critical items influencing future teachers’ AI adoption for ADHD education

#### 4.3.1 Perceived usefulness

The aim of this analysis is to examine the effect of different aspects of perceived usefulness on the intention to adopt artificial intelligence (AI) in an educational context, particularly for students with attention deficit hyperactivity disorder (ADHD). The analysis is based on a multiple linear regression, and the results show significant variations in how different perceptions of AI usefulness influence adoption.

The regression statistics indicate that the overall model is significant, with a multiple correlation coefficient of 0.547. The analysis reveals that perceived usefulness explains 29.89% of the variance in the intention to adopt AI (R^2^ = 0.299), suggesting a moderate but meaningful contribution of this variable in teachers’ decision-making process.

The results (Table 3) show that the item *“Using AI would make my teaching more effective for this group”* has the highest coefficient (*β* = 0.4343, *p <* 0.001). This suggests that future teachers primarily associate AI with pedagogical efficiency rather than as a tool for diagnosis or personalization.

**Table 3:** Factors related to the perceived usefulness of artificial intelligence in teaching students with ADHD.

Variable	Coefficient (*β*)	*P*-value
1. AI could help personalize learning for students with ADHD	0.2000	< 0.001
2. AI would improve my ability to identify the specific needs of students with ADHD	−0.0204	< 0.001
3. Using AI would make my teaching more effective for this group	0.4343	< 0.001

This perception is supported by qualitative interview data. One student teacher explained: *“As a future teacher, I know that time management is a challenge. If AI can help me prepare suitable materials more quickly and manage different levels better, it would be a real asset.”*

In contrast, the item “AI would improve my ability to identify the specific needs of students with ADHD” showed a negative and significant association with the intention to adopt AI (*β* = −0.0204, *p <* 0.001). This suggests that, among preservice teachers, those who believe AI can help identify students’ needs are paradoxically less inclined to adopt it. One possible interpretation is that this belief reflects anxiety about replacing human judgment in sensitive areas like assessment, rather than trust in AI capabilities. As one future teacher commented: *“Assessing a student is not just about analyzing data. There’s a human dimension, a relationship with the child. I find it hard to believe that an AI could understand a student better than a teacher.”*

This lack of confidence in AI for identifying specific needs may also reflect a broader need for training and guidance on existing tools. Unlike in-service teachers, future teachers have not yet experienced real classroom settings, which may increase their doubts regarding emerging technologies.

The item *“AI could help personalize learning for students with ADHD”* (Table 3) shows a moderate coefficient (*β* = 0.2000, *p <* 0.001), suggesting that future teachers recognize AI’s potential for pedagogical differentiation, although this is not the most decisive factor in their intention to adopt it. Some student teachers view AI as a support for adapting learning content, but express caution regarding the relevance of automated recommendations. One student in training explained: *“If AI suggests exercise ideas adapted to a student’s difficulties, that could help me — but human validation is always necessary. I want to keep control over my teaching.”*

These results indicate that future teachers see AI as a support tool but remain cautious about its role in student assessment and pedagogical decision-making. Their acceptance of such technologies depends on their familiarity with educational AI, which remains limited during initial teacher training.

#### 4.3.2 Perceived ease of use

The analysis of results related to perceived ease of use highlights differing effects of this variable’s components on the intention to adopt artificial intelligence (AI) for students with attention deficit hyperactivity disorder (ADHD). The multiple linear regression reveals that all items are significant (*p <* 0.001), confirming that perceptions of ease of use play a key role in the acceptance of these technological tools. Regression statistics indicate that the overall model is significant, with a multiple correlation coefficient of 0.513. The analysis shows that perceived ease of use accounts for 26.35% of the variance in the intention to adopt AI (R^2^ = 0.263), suggesting a moderate but meaningful contribution of this factor to teachers’ decision-making process.

The results (Table 4) show that interface intuitiveness is the strongest determinant of teachers’ intention to adopt AI tools. The item “*The interfaces of AI tools seem intuitive to me*” presents the highest coefficient (*β* = 0.3161, *p <* 0.001), suggesting that the ergonomic design of the tools is a critical factor in their acceptance. Some teachers expressed a clear preference for platforms with a clean and simple interface: “*I have tried several AI tools, and the ones with smooth, minimal interfaces are the ones I actually use. If it’s too complicated, I waste time and eventually give up*. «Another significant factor is the impact of AI on teachers’ workload. The item “*AI would not complicate my lesson planning”* received a coefficient of *β* = 0.2568 (*p <* 0.001), indicating that teachers assess the accessibility of AI tools based on the time and effort required to integrate them into their pedagogical practice. Several comments highlight that while AI can save time, it can also introduce complexity depending on the tool used: “*AI could help me generate teaching materials, but if it takes hours to learn how it works, it’s not viable.”“Some tools really do make lesson planning easier by suggesting exercises in seconds. Others require too much setup and become a burden*.”

**Table 4:** Factors related to the perceived ease of use of artificial intelligence in teaching students with ADHD.

Variable	Coefficient (*β*)	*P*-value
4. I would find it easy to learn how to use AI tools adapted for students with ADHD	0.1862	< 0.001
5. The interfaces of AI tools seem intuitive to me	0.3161	< 0.001
6. AI would not complicate my lesson planning	0.2568	< 0.001

Finally, the item *“I would find it easy to learn how to use AI tools adapted for students with ADHD”* shows a more moderate coefficient (*β* = 0.1862, *p <* 0.001). This suggests that most teachers believe they can learn how to use these tools, but that this perceived ability alone is not enough to ensure widespread adoption. Several participants pointed out that even if they feel capable of learning to use AI tools, actual use depends on whether the tools provide real added value in the classroom: *“I’m not afraid of learning how to use a new tool, but it has to be worth it and meet a concrete need.” “I’ve attended several AI training sessions, but if the tools aren’t designed to fit classroom realities, I will not use them.”*

These results confirm that perceived ease of use is a key factor in AI adoption, though not all of its dimensions carry the same weight. Interface intuitiveness emerges as the strongest driver of acceptance, followed by the impact of AI on workload, while teachers’ confidence in their ability to learn plays a secondary role. These findings highlight the need for ergonomic tool design and gradual integration into teaching practices, supported by appropriate training programs.

#### 4.3.3 Subjective norms

The analysis of results related to subjective norms (Table 5) reveals a significant impact of these factors on teachers’ intention to adopt artificial intelligence (AI). All variables included in the regression model are statistically significant (*p <* 0.001), confirming that institutional recommendations and the professional environment play a role in the decision to integrate AI into teaching practices. However, the strength of this impact varies depending on the perceived source of influence.

**Table 5:** Factors related to subjective norms and social influence of artificial intelligence in teaching students with ADHD.

Variable	Coefficient (*β*)	*P*-value
7. My teacher educators recommend using AI for students with ADHD	0.3641	< 0.001
8. Experienced teachers around me use or recommend AI	0.1111	< 0.001
9. My training institution encourages technological innovation in the classroom	0.1862	< 0.001

Regression statistics indicate that the overall model is significant, with a multiple correlation coefficient of 0.484. The analysis shows that subjective norms and social influence explain 23.45% of the variance in the intention to adopt AI (R^2^ = 0.234), suggesting a moderate but meaningful role of these factors in teachers’ decision-making process.

The results (Table 5) indicate that the role of teacher educators is the primary driver of AI adoption among future teachers. The item *“My teacher educators recommend using AI for students with ADHD”* has the highest coefficient (*β* = 0.3641, *p <* 0.001), suggesting that pedagogical experts play a decisive role in encouraging teachers to integrate AI into their practice. Several participants reinforced this trend by emphasizing the role of trainers as mediators between the technology and its classroom application: *“When my trainer showed us concrete examples of using AI with students with ADHD, it made me want to try. Without their expertise, I probably would not have dared to get started.” “I trust specialized trainers because they understand our needs and the realities of the classroom. Their recommendations carry more weight than any advertisement or institutional directive.*

The institutional environment also plays a role, although to a lesser extent. The item *“My training institution encourages technological innovation in the classroom”* has a coefficient of *β* = 0.1862 (*p <* 0.001), suggesting that institutional initiatives can support AI adoption, but are not decisive on their own. Institutions that promote innovation may create a favorable climate for experimentation, but this influence appears less direct than that of trainers. As one student teacher explained: *My institution promotes new technologies, but without concrete support, many teachers remain skeptical. It’s mainly the trainers who help us understand how to use these tools effectively.”* Another added: *“The administration encourages us to test AI, but it’s the exchanges with trainers and colleagues that really influence my decision to adopt these tools.”*

Finally, the influence of experienced colleagues appears to be the least impactful factor in AI adoption. The item *“Experienced teachers around me use or recommend AI”* shows the lowest coefficient (*β* = 0.1111, *p <* 0.001), though still statistically significant. This suggests that while peers can influence attitudes, their impact is more limited compared to that of trainers and institutions. Some teachers explained this by pointing to the diversity of practices and comfort levels with technology among colleagues: *“Some colleagues are enthusiastic about AI, others are more hesitant. It’s not their opinion that will convince me, but rather targeted training.”*

These findings confirm that social norms and institutional recommendations play a role in AI acceptance among teachers, though the impact varies depending on the source of perceived influence. Support from specialized trainers appears to be the most effective lever for promoting the integration of these technologies, while the influence of peers and institutional frameworks remains more moderate.

#### 4.3.4 Social influence

The analysis of the effects of social influence on teachers’ intention to adopt artificial intelligence (AI) highlights the significant role of perceptions related to professional image and recognition. Results from the multiple linear regression show that both items analyzed are statistically significant (*p <* 0.001), confirming that the social dimension of technology acceptance plays a key role in shaping teachers’ willingness to integrate AI into their pedagogical practices.

Regression statistics indicate that the overall model is significant, with a multiple correlation coefficient of 0.578. The analysis reveals that social influence accounts for 33.35% of the variance in the intention to adopt AI (R^2^ = 0.334), suggesting a substantial contribution of this variable to teachers’ decision-making process.

The results (Table 6) indicate that the social value placed on innovation plays a key role in future teachers’ decision to adopt artificial intelligence (AI) in their practice. The item *“Using AI would strengthen my credibility as an innovative teacher”* shows the highest coefficient (*β* = 0.418, *p <* 0.001), suggesting that AI adoption is perceived as a marker of distinction and professional recognition. Several participant statements reflect this perception:

**Table 6:** Factors related to social influence of artificial intelligence in teaching students with ADHD.

Variable	Coefficient (*β*)	*P*-value
10. Using AI would strengthen my credibility as an innovative teacher	0.418	< 0.001
11. AI tools are perceived as modern in the educational field	0.313	< 0.001

*“As a future teacher, I want to stand out and show that I’m at the forefront of new teaching practices. AI can be an asset for that.” “I feel like teachers who master AI are better perceived by colleagues and employers. It gives an image of professionalism and innovation.”*

The association between AI and modernity in education also influences adoption intentions, though to a lesser extent. The item *“AI tools are perceived as modern in the educational field”* has a significant but more moderate coefficient (*β* = 0.313, *p <* 0.001). This suggests that while future teachers recognize the innovative image associated with AI, this perception is less influential than the personal value placed on being seen as an innovator. As some participants explained: *“AI is becoming more present in pedagogical discussions. If we want to stay aligned with new methods, we cannot ignore it.” “We hear a lot about innovation in training, and AI is one of the technologies seen as the future of education.”*

However, several future teachers expressed reservations about using AI solely for its image-enhancing potential: *“I do not want to use AI just to look innovative. It has to be truly useful and bring something to the students.” “Just because a tool is modern does not mean it’s effective. I need to see real results before I decide to adopt it.”*

These findings suggest that the social acceptability of AI is a central lever for its adoption in education. The more teachers perceive AI as a way to enhance their professional status and recognition as innovative practitioners, the more likely they are to adopt it.

#### 4.3.5 Experience and willingness

The analysis of the effects of prior experience with technology and willingness to engage with AI reveals contrasting trends in teachers’ acceptance of artificial intelligence. The multiple linear regression indicates that the overall model is significant, with a multiple correlation coefficient of 0.449. The analysis shows that experience and willingness explain 20.12% of the variance in the intention to adopt AI (R^2^ = 0.201), suggesting a moderate contribution of these variables to teachers “decision-making process.”

Both items analyzed are statistically significant (*p <* 0.001), indicating that these dimensions influence the intention to adopt AI in different ways.

The results reveal (Table 7) that the institutional framework plays a key role in the acceptance of AI among future teachers. The item *“If AI were required by my institution, I would still find it useful”* shows the highest coefficient (*β* = 0.424, *p <* 0.001), indicating that the adoption of AI tools can be strongly related to institutional directives. This suggests that even initially skeptical teachers may come to view AI as useful if its use becomes a standard within their institution. Testimonies from future teachers confirm this trend:

**Table 7:** Factors related to experience and willingness toward artificial intelligence in teaching students with ADHD.

Variable	Coefficient (*β*)	*P*-value
12. I have already used technological tools similar to AI (e.g., adaptive software)	0.119	< 0.001
13. If AI were required by my institution, I would still find it useful	0.424	< 0.001

*“If AI becomes a requirement at my institution, I will take the time to learn it, even if I wasn’t convinced at first.” “When a tool becomes mandatory, we often adopt it out of necessity. Later, we might realize it’s actually useful.”*

This institutional influence highlights the importance of organizational support in facilitating the adoption of educational technologies. A structured framework and gradual integration appear to be key factors in fostering acceptance among future teachers: *“If we are clearly told why AI is useful and how to integrate it into our teaching, I’ll be much more likely to adopt it.”*

In contrast, prior experience with educational technologies similar to AI appears to have a more limited impact. The item “*I have already used technological tools similar to* AI (e.g., adaptive software)” shows a lower coefficient (*β* = 0.119, *p <* 0.001), suggesting that familiarity with other educational tools does not automatically lead to the acceptance of AI. Some student teachers expressed this idea clearly: *“I’ve used adaptive software, but AI feels like another level. I still struggle to see how to use it effectively in the classroom.”*

*“Just because we have used digital tools before does not mean we are ready to adopt AI. We need real training and a clear understanding of its value.”*

These findings underscore that AI acceptance in educational settings is strongly shaped by institutional structures and policy guidance. Institutional mandates can encourage broader adoption, even among initially hesitant teachers, whereas prior experience with digital tools alone is not a decisive factor. 416.

## 5 Discussion

### 5.1 Factors affecting the adoption and acceptability of AI in pedagogical practice

The main objective of this study was to examine the factors that shape the acceptability of AI tools in teaching students with ADHD.

Based on the regression coefficients (*β*), the results confirm that perceived usefulness (PU) and perceived ease of use (PEU) are key factors in teachers’ acceptance of AI.

The integration of AI into education opens new opportunities to improve the effectiveness and relevance of teaching practices (Hernes and UNESCO, 2002), while also reducing the burden of many daily tasks for teachers (Jabraoui and Vandapuye, 2024). In particular, AI helps reduce administrative workload, allowing teachers to dedicate more time to individualized student support (Ido, 2016).

Today, AI is emerging as a powerful pedagogical support tool, facilitating the identification of learning difficulties, real-time progress monitoring, as well as assessment and instructional adaptation (Senate–Delegation for Forward Planning et al., 2024). The relevance of this functional dimension is also illustrated in the study by Smith et al. (2023), *Exploring the Role of Artificial Intelligence in Education: Toward a Personalized Learning Experience*. This research draws on several case studies to analyze how AI is integrated into the curricula of various educational institutions. The findings show notable improvements in learner engagement and performance, highlighting AI’s contribution to optimizing teaching processes and personalizing learning.

In the specific context of teaching students with ADHD, these effects are even more pronounced. Teachers are more likely to use AI when it is perceived as easy to use and beneficial for differentiated, learning. This trend aligns with the findings of Barua et al. (2022) and Moleka (2023), who highlight the concrete benefits of AI in adapting content, pace, and teaching methods to the specific needs of these learners, thereby contributing to greater school inclusion.

Furthermore, innovations such as the virtual robotic agent VACO, described by Richter et al. (2023), and various mobile applications identified by Kyriakaki and Driga (2023), have been recognized as promising tools for improving attention and time management among students with ADHD.

Beyond usefulness and ease of use, other variables also play a key role in AI adoption by teachers—particularly prior experience with technology and willingness to adopt. Previous use of educational technologies and a proactive attitude toward trying new tools is significant factors. Teachers who are familiar with digital tools or who express a willingness to integrate AI are more likely to adopt it. This observation is supported by Cukurova et al. (2023), who found that teachers’ confidence in using adaptive learning platforms and their openness to these tools positively influence their actual engagement.

In this perspective, experience and willingness cannot develop without a supportive institutional environment. To enable teachers to fully benefit from the advantages of AI, it is essential to implement targeted training programs. These should address both the technical and pedagogical skills needed for effective integration. This need echoes the conclusions of Jabraoui and Vandapuye (2024), who emphasize that AI adoption requires significant adjustment time and access to appropriate resources. Without structured support, these technologies may be perceived as complex or time-consuming, becoming a major barrier to long-term integration in teaching practices. Therefore, strong institutional support is crucial to turn individual willingness into real engagement.

In addition, social influence and subjective norms also play a role, although less prominently. The study by Menant (2021) on the acceptability of AI in human resources information systems shows that perceptions and encouragement from the professional environment, as well as social norms related to AI use, can affect the intention to adopt. However, their impact remains lower than that of perceived usefulness and individual experience.

### 5.2 Influential variables within each acceptance factor

The results of this study highlight several factors that influence future teachers’ intention to adopt artificial intelligence (AI). The analysis of regression coefficients and the qualitative data collected during interviews confirm that perceived usefulness is the main determinant of AI acceptability in the educational context.

The most influential item within perceived usefulness is the belief that AI would enhance teachers’ instructional effectiveness. This trend is supported by interview responses, where many participants noted that AI could help manage administrative tasks and improve the adaptation of instructional materials to students’ needs. This result aligns with existing theories on educational technology acceptance, especially the Technology Acceptance Model (TAM), which emphasizes that perceived usefulness is the primary predictor of technology adoption (Venkatesh and Davis, 2000).

Regarding perceived ease of use, the results suggest that interface intuitiveness plays a crucial role in AI acceptability. The more teachers perceive tools as accessible and easy to use, the more likely they are to integrate them into their teaching. This finding is consistent with technology acceptance models (Davis, 1989; Venkatesh and Davis, 2000), which state that ease of use directly impacts the intention to adopt new technologies. These results highlight the importance of developing user-friendly interfaces tailored to teachers’ needs to promote broader adoption of AI tools in educational settings.

The analysis of subjective norms and social influence shows that the opinions of specialized teacher educators are a determining factor in the acceptance of AI. Future teachers are more inclined to integrate AI into their practice when it is promoted by pedagogical experts who are viewed as credible sources of guidance and support. This finding is consistent with previous research showing that the adoption of educational innovations is often encouraged by the influence of experts and trainers (Fishbein and Ajzen, 1975; Venkatesh and Bala, 2008). Therefore, to foster the effective adoption of AI by future teachers, it is essential to integrate these technologies into teacher training programs, along with clear recommendations from educational trainers.

Social influence also plays a significant role in the intention to adopt AI. The item indicating that social recognition of innovation is a key factor in the decision to use AI shows the strongest effect. This finding aligns with technology acceptance theories, particularly the social influence model developed by Venkatesh and Davis (2000), which emphasizes that users are more likely to adopt a technology when it is perceived as a source of professional recognition. In the educational context, this suggests that AI is not only evaluated for its pedagogical benefits, but also as a distinguishing factor that allows teachers to be seen as innovative and forward-thinking by their peers and superiors.

Finally, the analysis of experience and willingness shows that AI adoption can be strongly related to institutional directives. A significant proportion of participants stated that *“if AI were required by my institution, I would still find it useful,”* indicating that alignment with organizational expectations plays a central role in the acceptance of educational technologies. This phenomenon is consistent with technology acceptance models (Venkatesh and Davis, 2000), which suggest that institutional requirements can drive adoption, even in the absence of initial commitment. These results highlight the importance of a structured organizational framework in implementing educational technologies: clear and supportive institutional guidance can enhance AI acceptability and encourage its gradual integration into pedagogical practices.

Several past and recent studies have highlighted the benefits of AI in the context of inclusive education, especially for students with attention disorders, including ADHD. Building on this literature, our research confirms that AI can significantly support adaptive learning for these students. These findings open several promising research avenues. First, it is essential to develop adaptive learning software specifically designed for students with ADHD, enabling real-world classroom experimentation. Next, implementing longitudinal studies would help assess the medium- and long-term impact of AI on both academic and behavioral development in this population. Additionally, expanding research to include other neurodevelopmental disorders would provide a clearer understanding of AI’s potential in achieving truly inclusive education. Finally, establishing a clear ethical and regulatory framework, alongside the deployment of continuous professional training for teachers, appears crucial for the responsible, equitable, and sustainable integration of AI into educational practices.

## 6 Limitations of the study

This study presents several limitations that must be considered when interpreting the findings. First, in terms of generalizability, the sample was composed exclusively of preservice teachers from a single teacher training institution in Morocco. As such, the results may not be representative of other cultural, institutional, or educational contexts. Second, the study used a cross-sectional design based on correlational analysis, which does not allow for causal inferences regarding the relationships between the examined variables. Future research employing longitudinal or experimental designs would be needed to assess how these factors evolve over time or affect actual behavior. Third, the use of self-reported questionnaires, although based on validated constructs from the TAM2 model, introduces potential biases such as social desirability or subjective interpretation of items. In addition, some constructs were measured with a limited number of items (two to three), which may reduce the depth and robustness of measurement. Despite acceptable reliability scores, further research should explore more comprehensive and diversified tools to assess technology acceptance in educational contexts.

## 7 Conclusion

This study aimed to explore the factors that influence the acceptability of artificial intelligence (AI) among future teachers in the context of teaching students with attention deficit hyperactivity disorder (ADHD). Using the TAM 2 model as a theoretical framework and a mixed-methods approach, results show that perceived usefulness is the most influential factor in the intention to adopt AI, followed by perceived ease of use, willingness, and finally social and institutional influences. The analysis also revealed that specific items—such as interface intuitiveness and pedagogical trainers’ recommendations—play a decisive role in the decision-making process. These findings confirm that AI adoption among pre-service teachers does not depend solely on the technical features of the tools. It is also shaped by contextual, institutional, and symbolic factors. AI is perceived as an opportunity to personalize learning and enhance pedagogical effectiveness—provided it is intuitive, supported by clear guidance, and integrated into a coherent training strategy. In this regard, teacher education institutions play a critical role in fostering a climate that supports the appropriation of emerging technologies, particularly through initial and continuing training programs focused on the pedagogical uses of AI and the management of special educational needs.

Finally, this study opens up several avenues for future research. It is essential to develop AI tool specifically tailored to students with ADHD, through close collaboration between researchers, teachers, and developers. Moreover, longitudinal studies are needed to evaluate the long-term impact of AI on these students’ learning trajectories and on teachers’ professional practices. Expanding this research to other neurodevelopmental disorders would provide a deeper understanding of AI’s potential in inclusive and differentiated education. Lastly, the establishment of a clear ethical and regulatory framework—ensuring equity, transparency, data protection, and non-discrimination—is a necessary prerequisite for any large-scale implementation of these technologies in educational settings.

## Data Availability

The raw data supporting the conclusions of this article will be made available by the authors, without undue reservation.
